# 

**Published:** 1901-05-18

**Authors:** 


### NOTICE TO CORRESPONDENTS.

All MSS., Letters, Books for Review, and other matters intended for the Editor should be addressed The Editor, "The Hospital," The Hospital Building, 28 & 29 Southampton Street, Strand, W.C.

The Editor cannot undertake to return rejected MS., even when accompanied by stamped directed envelope.

**Notice to Advertisers and Subscribers.**

All Advertisements, Orders for Copies of The Hospital, and Business Communications should be addressed to **THE MANAGER (Not to the Editor),** The Hospital, 28 & 29 Southampton Street, Strand, London, W.C.

The Cost of Subscription, post free, is as follows:—Per week, 2½d.; per quarter, 2s. 8d.; per half-year, 5s. 3d.; per annum, 10s. 6d. Foreign:—Per annum, 15s. 2d., payable, in all cases, in advance.

**NOTICE.—Vol. XXIX. of "The Hospital" (October 1900, to March, 1901), in handsome cloth binding are now on sale at the Office of this Journal, price, 7s. 6d. Cases for binding the Numbers contained in this Volume 1s. 6d. Index to Vol. XXIX., 2½d., post free.**

**The Monthly Part for May is now ready. Price 6d., by post 8d.**

### SCRAPS AND GLEANINGS.

THE bazaar held on behalf of the Bristol Children's Hospital realised a nett sum of £2,500.

DURING the year the total number of accidents treated at the Cork Northern Charitable Infirmary was 7,520; and the number of extern-cases was 28,000.

LAST year's Bolton Hospital Saturday collection realised the sum of £3,254. This collection was instituted twenty years ago, and the result of the first year's effort was £600.

THE Ropner Convalescent Home has recently been opened for the season, when 22 patients took up their quarters there. There is altogether accommodation for 62 patients in the home.

DURING the year 1900 the ordinary income of the Inverness Northern Infirmary was £2,450 as compared with £2,816 the previous year. The ordinary expenditure was £4,611, and legacies amounted to £1,500.

AT the annual meeting of the Suffolk Convalescent Home, Felixstowe, it was stated that 444 patients were admitted during the year, and that since the home had been established in 1868 there had been 9,645 admissions.

DURING the past year the ordinary subscriptions to the Newcastle Infirmary amounted to £3,708; the donations totalled £1,525; the contributions from workmen £4,900, and the church and chapel collections £1,410. The ordinary expenses of the year amounted to over £16,500.

DURING the eight years that Hospital Saturday has been established at Guildford the sum of £1,309 has been collected. Of this sum the Royal Surrey County Hospital had received £826, the Surrey Convalescent Home for Men £347, and the Victorian Home for Surrey Women £58.

SATURDAY, May 25th, has been fixed for the Hospital Saturday collection on behalf of the Belfast Maternity Hospital. This is the first Saturday collection on behalf of this hospital, and in order that it may prove a success the promoters of the Royal Hospital Saturday collection have postponed their collection to the first Saturday in September.

AN application has been made to the Local Government Board for sanction to borrow £5,510 for the extension of the Infectious Diseases Hospital at Clatterbridge. This sum is to be utilised in paying for the land required, viz., £600; and for the erection of a new ward to contain 16 beds, an isolation ward to contain four beds, three bedrooms, and lavatory accommodation.

THE architect's plans for the new Workhouse Hospital at Halifax provide for accommodation for 642 patients, but, for the present, accommodation for only 400 beds has actually been supplied in the newly-erected buildings, which are so arranged that additional beds can be provided by the addition of one or two pavilions, as may be required. The total estimated cost of the buildings is about £130,000.

At a recent meeting of the Wisbech Hospital trustees and committee the report and recommendations of the sub-committee appointed to consider the expediency of establishing an outpatient department in connection with the hospital was adopted, and the scheme ordered to be submitted to the subscribers. It was stated at the meeting that the scheme would be opposed by the medical men on the ground that it was not required.

The recommendations of the Committee which has been considering the proposed Victoria Memorial for Maryport are shortly to be submitted to a public meeting. The Committee recommends the erection of a cottage hospital with two wards, each providing for four patients, a separation room with accommodation for one patient, and the necessary administration rooms. The cost of the buildings and furnishing is estimated at £1,300, and the annual cost of maintenance is estimated at £280.

### The Student of Medicine.

### APPOINTMENTS.

**H. T. Barron**, M.D.Lond, M.R.C.S., L.R.C.P., appointed Clinical Assistant to the Royal Eye Hospital, Southwark. **Miss Agnes Blackadder**, M.D., appointed Assistant Medical Officer for the Smithson Road Workhouse of the Toxteth Union. **G. Bower**, L.R.C.P., L.R.C.S.Edin., appointed Medical Officer of Health for the Sutton Urban District. **J. J. Boyd**, M.B., Ch.M.Glasg., D.P.H.Camb., appointed Medical Officer of Health for South Shields. **Elmore Brewerton**, F.R.C.S.Eng., appointed Ophthalmic Surgeon to the Metropolitan Hospital. **Arthur Connell**, M.R.C S.Eng., L.R.C.P.Lond., F.R.C.S.Edin., appointed Honorary Surgeon to the Royal Infirmary, Sheffield. **Herbert Chessons**, M.R.C.S.Eng., L.R.C.P.Lond., appointed Assistant Medical Superintendent of the Hospital for the Insane at Goodna, Queensland. **D. R. Powell Evans**, L.R.C.P.Lond., M.R.C.S.Eng., L.S.A.Lond., appointed Public Vaccinator for the North Wimbledon District of the Kingston Union. **Maude E. Fendick**, L.S.A., appointed Assistant House Surgeon to the North Riding Infirmary, Middlesbrough. **W. Finlay**, M.D., appointed Health Officer for the Shire of Tungamah, Victoria. **Alexander Galloway**, L.R.C.P., L.R.C S.Edin., L.F.P.S.Glasg., appointed Medical Officer to the South Shields District of the South Shields Union. **O. F. Gmelin**, M.D., appointed Health Officer for the Shire of Eltham, Victoria. **Randolph Grosvenor**, B.A.Cantab., L.R.C.P.Lond., M.R.C.S., appointed Honorary Physician to the Chelsea, Brompton, and Belgrave Dispensary, Sloane Square, S.W. **Frances Ivens**, M.B.Lond., appointed Clinical Pathologist and Assistant Pathologist to the Royal Free Hospital, London. **Sydney Jamieson**, M.B., Ch.M.Edin., L.R.C.P., appointed Lecturer on Pathology at the University of Sydney. **J. T. C. Nash**, M.D., D.P.H., appointed Acting Professor of Hygiene at King's College, London, during the absence of Professor Simpson in South Africa. **V. O'Reilly**, M.B., B.Ch.R.U.I., appointed Medical Officer of Health for the Skelmersdale Urban District. **Fred. W. Pollard**, B.A., Lic. Med. Surg. and Mid. Univ. Dublin, appointed Medical Officer and Public Vaccinator, No. 2 district, Blackburn Union. **Frederick W. Price**, M.B., C.M.Edin., appointed Pathologist to the City of London Hospital for Diseases of the Chest, Victoria Park. **Arthur Pring**, L.R.C.P., appointed Medical Officer at Beaudesert. **Clarence Read**, M.R.C.S., L.R.C.P., appointed Honorary Medical Officer to the North Shore Hospital, Sydney. **George Edward Rennie**, B.A.Syd., M.D., M.R.C.P. Lond., appointed Tutor in Medicine in the University of Sydney, N.S.W. **W. E. Robinson**, M.R.C.S., L.R.C.P., appointed Medical Officer and Public Vaccinator for the Sonning District of the Wokingham Union. **W. A. Spring**, appointed Health Officer for the Shire of Traralgon, Victoria. **H. C. Sturdy**, M.B., B.S., appointed Junior House-Physician to the City of London Hospital for Diseases of the Chest, Victoria Park. **Abraham George Sutton**, L.R.C.P., L.R.C.S.Edin., appointed Resident Medical Officer to the Cork Fever Hospital. **W. C. Wilkinson**, M.D., appointed Lecturer on Medicine at the University of Sydney. **Oliver K. Williamsom**, M.A., M.B., B.C.Cantab., M R.C.P.-Lond., appointed Physician to the Out-patients at the City of London Hospital for Diseases of the Chest, Victoria Park.

